# Training in Pediatric Surgical Oncology

**DOI:** 10.3389/fped.2022.848543

**Published:** 2022-03-21

**Authors:** Paul D. Losty

**Affiliations:** Department of Paediatric Surgery, Institute of Life Course and Medical Sciences, University of Liverpool, Liverpool, United Kingdom

**Keywords:** pediatric surgery, surgical training, surgical oncology, innovation, university

## Abstract

Pediatric Surgical Oncology is a challenging sub-speciality field requiring strong discipline, resilience and a “never quit” attitude. Pediatric cancer patients are frail, vulnerable and have life limiting illness were the skill(s) of the operating surgeon may determine “cure” and survival or untimely death from inadequate tumor resection with disease progression and / or “never event” misadventure. It could be stated therefore that a career in surgical oncology may not be for the “faint hearted” surgeon. Training in a high volume center(s) accredited in the delivery of high quality care and clinical excellence driven by inspirational leaders in surgical oncology with motivated multidisciplinary teams is key. International surgery oncology fellowship(s) add significant credits to a residents' clinical training crucially also yielding opportunities for gaining research skills. On the journey toward subspeciality accreditation the aspiring surgeon oncologist must first demonstrate skill set(s) proficiency in general pediatric surgery residency training to advance to the next phase(s). This article offers a “snap shot” overview synopsis of pediatric surgical oncology training in its broadest perspective(s) for those seeking further information and career guidance.

“*You Only Train Once?” ….*.

## The Start of the Journey—Training in Surgical Oncology—A Personal Story

**Figure 1:**
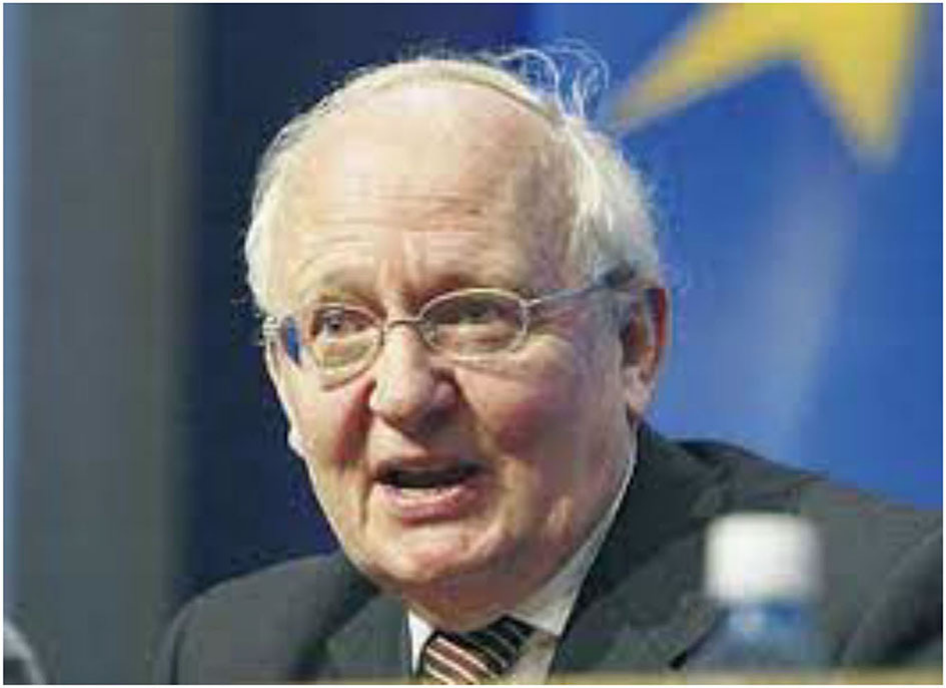
Professor Niall O'Higgins MCh FRCS—St Vincent's Hospital, University College Dublin, Ireland.

**Figure 2:**
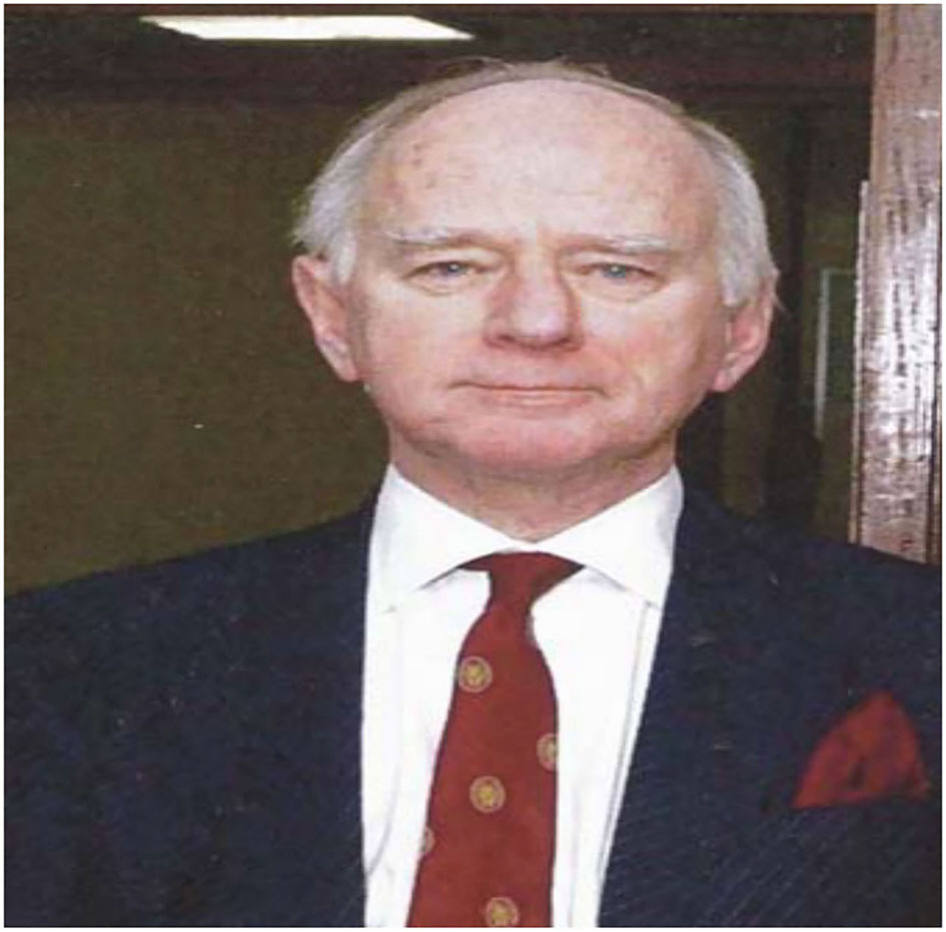
Professor Ray Fitzgerald MA FRCS FRACS—Trinity College Dublin, Ireland.

**Figure 3:**
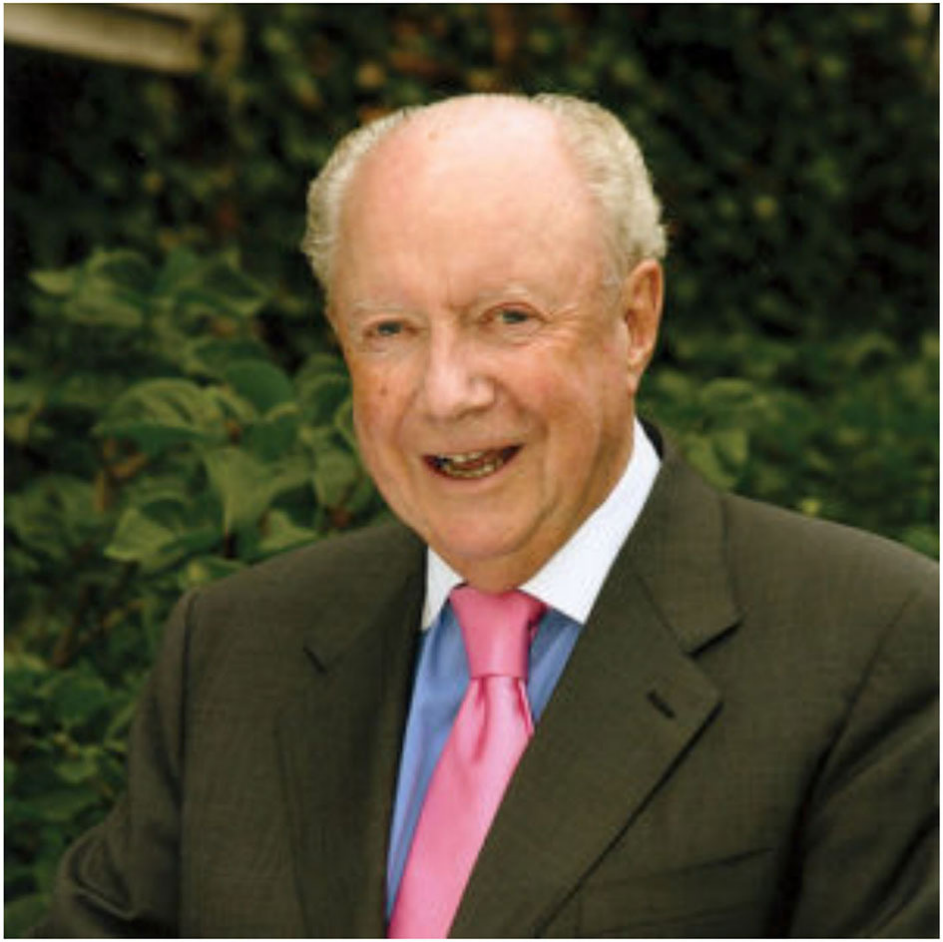
Professor Barry O'Donnell MCh FRCS—Royal College of Surgeons in Ireland.

**Figure 4:**
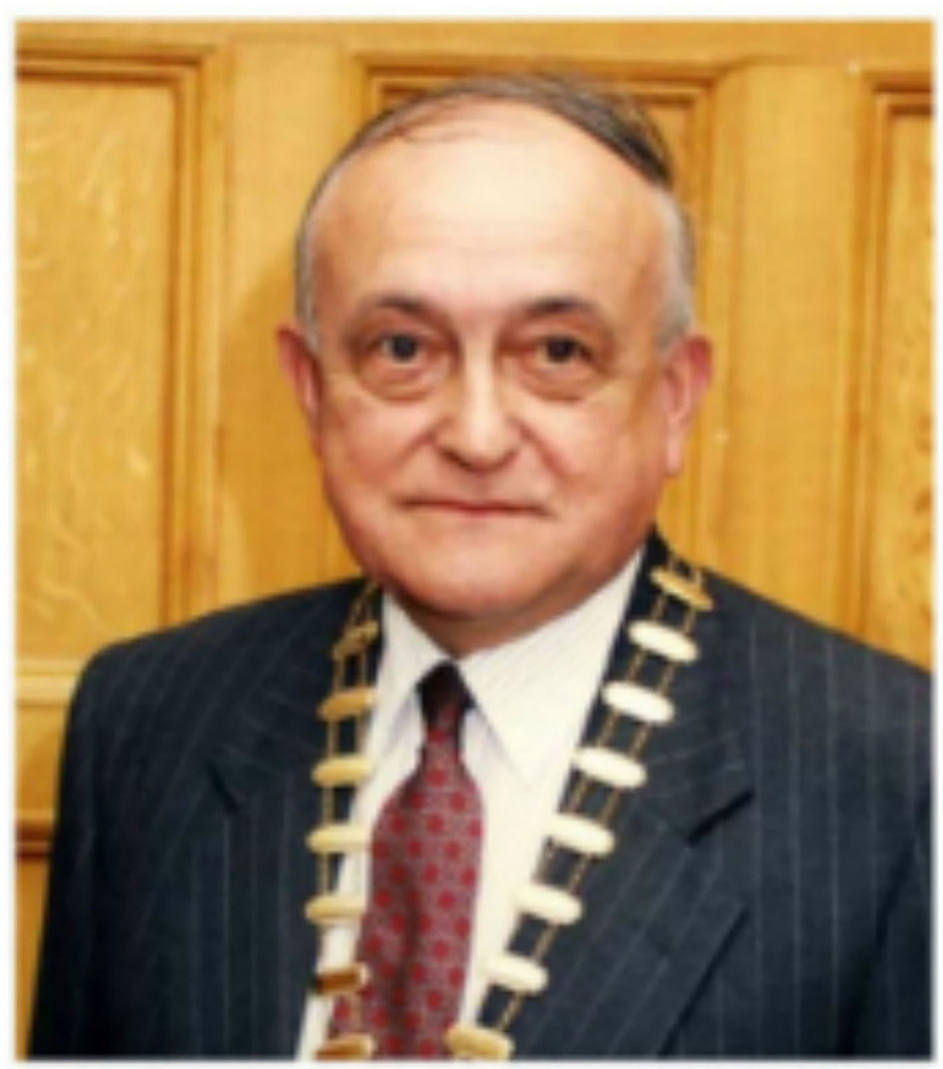
Professor Edward J. Guiney MCh FRCS—University College Dublin, Ireland.

**Figure 5:**
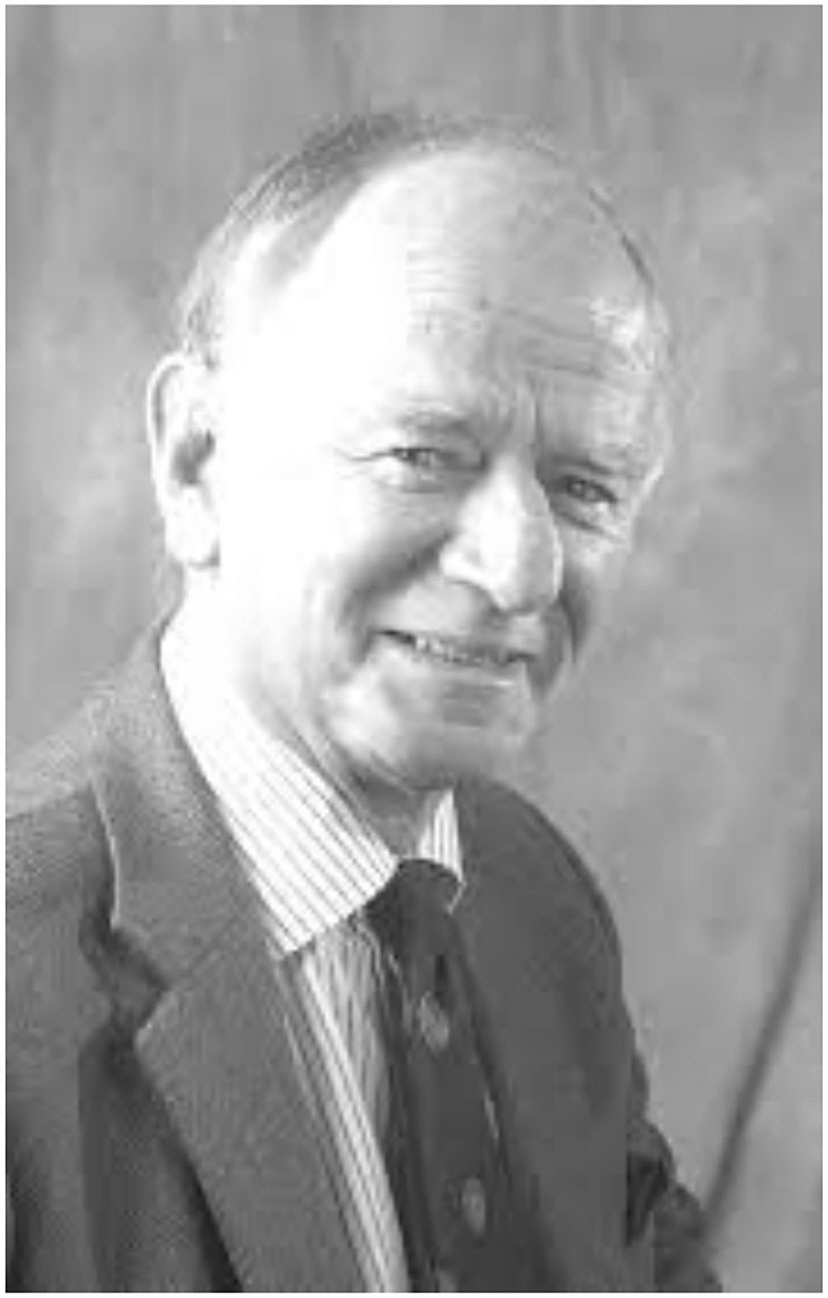
Professor Ray Fitzgerald MA FRCS FRACS—Trinity College Dublin, Ireland.

**Figure 6:**
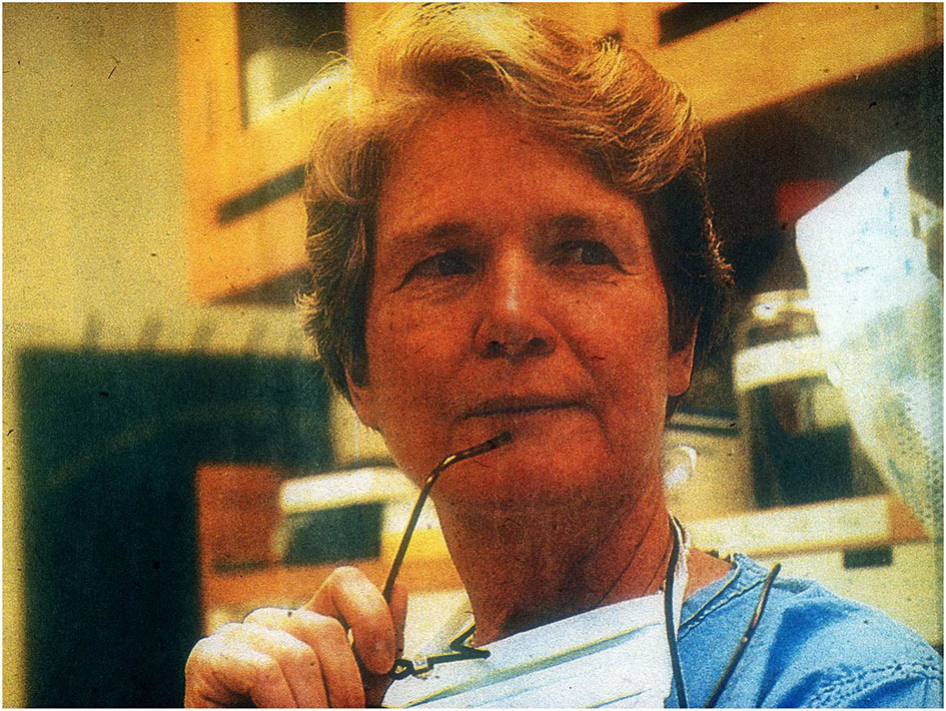
Professor Patricia Donahoe MD—Chief Of Pediatric Surgery, Massachussetts General Hospital, Harvard Medical School, Boston USA.

Getting started or wishing to train as a pediatric surgical oncologist is often influenced by exposure to leaders and role models during early surgical residency. My own personal journey was greatly influenced by working in a university department adult surgical oncology service led by an inspiring Professor of Surgery Niall O'Higgins at University College Dublin, Ireland—Figure 1. The clinical service was always busy with a high case load volume, several major operating lists weekly and many excellent regularly held department academic meetings. Training was a joy and privilege in these early years at St Vincent's Hospital, Dublin, Ireland. Rotating to a Senior House Officer (SHO) post in Pediatric Surgery on the Dublin RCSI Training Scheme I was then fortunate to work with Ray Fitzgerald at The Children's Hospital Temple Street, Dublin who was a technically superb surgeon setting very “high standards” expected of his team and a demanding chief likely rooted from his early Royal Navy career i.e.,—“Get It Right First Time”—Figure 2. Residency in Pediatric Surgery in Dublin, Ireland then followed after obtaining the FRCS returning to work with Ray Fitzgerald, Barry O'Donnell and Edward Guiney—Figures 3–5. Securing a surgeon faculty post at Alder Hey Children's Hospital and University Of Liverpool transpired in the early 1990's after having completing a “much sought after” surgical research fellowship at the Massachusetts General Hospital at Harvard Medical School with Professor Pat Donahoe—Figure 6.

During these formative years along with completing a residency in Pediatric Surgery I had trained in General Surgery, Vascular Surgery, Thoracic Surgery, Plastic Surgery and Trauma with many great surgeons - I firmly decided I was going to be a university pediatric surgeon with a subspeciality oncology practice. It may be said here at this point that an academic pediatric surgeon can enjoy the benefit(s) of a rewarding career where clinical job diaries with “surgical research time” are contracted and honored accordingly allowing a perfect match for surgical oncology.

## So What Skills Are Required?

Young surgeon(s) must be wholly mindful of the landscape in their own nations if they wish to train in pediatric surgical oncology. Where are the busy “high volume” surgery oncology centers where you can get and be assured of surgical “hands on” training? Be cognisant that pediatric cancer whilst the second leading cause of death in childhood in many developed world nations remains a rare disease and index cases notably solid tumor malignancies such as Wilms tumor (80 new cases/annum in the UK—CCLG) and neuroblastoma (100 new cases/annum in the UK—CCLG) are greatly surpassed by hematological cancers and CNS tumors. You will not want to work therefore in a surgery oncology center with a low index case load volume of solid tumors split amongst several residents and the consultant staff.

**Figure 7:**
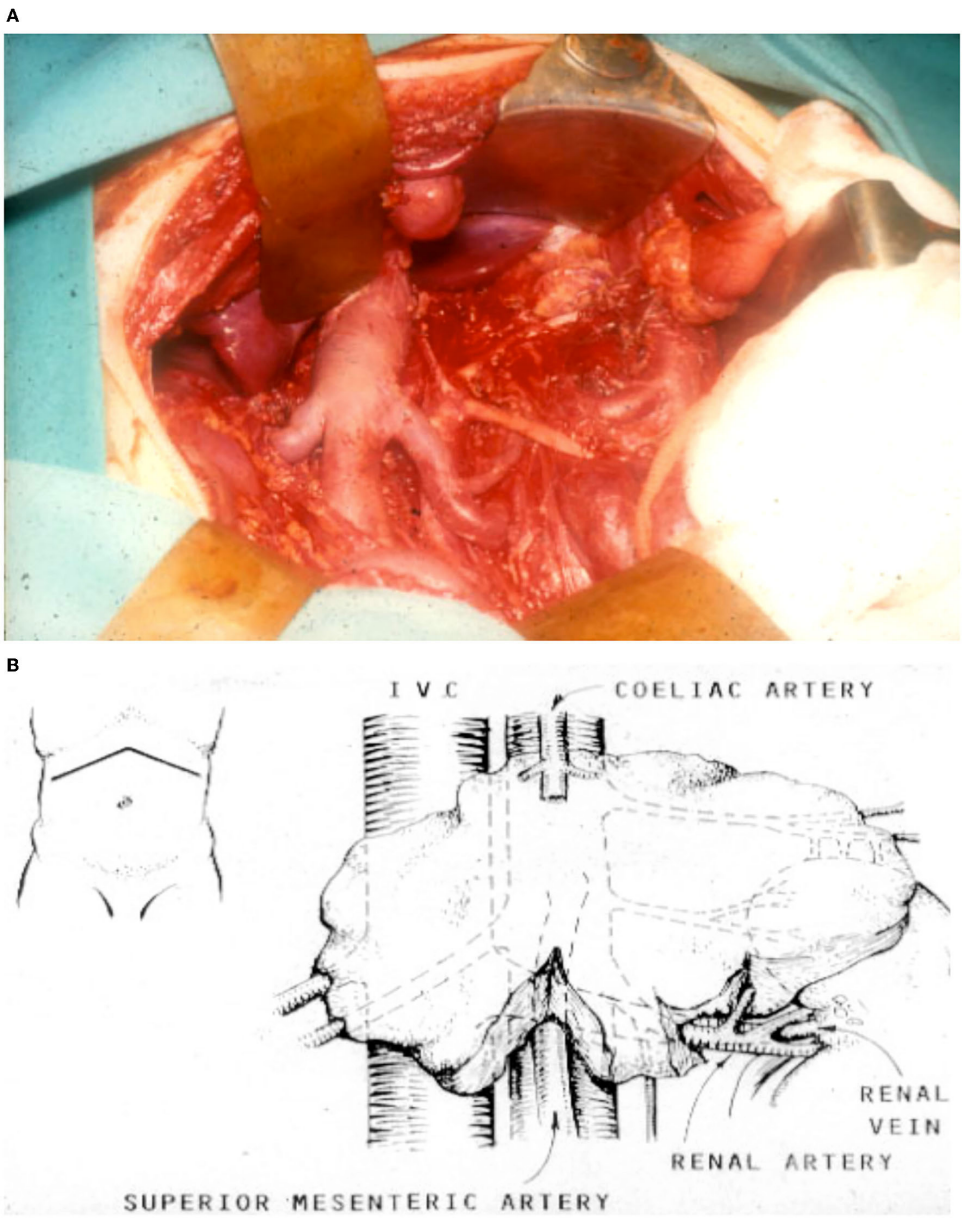
**(A)** Operative field photograph following gross total resection of an abdominal neuroblastoma tumor. **(B)** Schematic image of neuroblastoma tumor. Note a precise and painstaking careful skeletonization of major vasculature is required for a successful operation.

A surgical skills set acquired by spending attachements in vascular, plastic and thoracic surgery departments are invaluable (including urology) prior to a pediatric surgery residency programme before advancing to become a surgical oncologist. Technical expertise acquired in the “basic principles of surgery” here cannot be overemphasized. Surgical oncology demands the operator to have capabilities in a wide range of techniques e.g., vascular access, vessel dissection, isolation and control, vascular suture repair, reconstruction, organ sparing procedures and precision tissue handling—Figure 7.

Get busy then with your search when applying for these highly competitive surgery posts. At this stage if truly vocationally committed to pediatric surgical oncology also consider international fellowship training (see later). Ask yourself also what research is going on in these leading surgical centers? Will it be profitable for you? Is it exciting and worthwhile to explore? Crucially what is the track record of the surgical department(s) and their faculty staff? Do they inspire? What do staff (past & present) say about them? Are the faculty national and international leaders in their field? Do your homework. Choose (if you can) what best “fits for you.”

## International Surgical Oncology Fellowships

Having completed pediatric surgery training hopefully in a busy hospital programme or consortium hospital network partnership with exposure to surgical oncology do then consider further opportunities with advanced progression to seeking out a surgical oncology fellowship. Currently the best programmes available for international medical graduates are located in North America. Memorial Sloan Kettering Cancer Center, New York, USA and St Jude's Hospital Memphis Tennessee for example publicize fellowship positions regularly in the major pediatric surgical journals including with other professional organizations such as the International Society Of Pediatric Surgical Oncology (IPSO). It is greatly hoped in the near future further fellowships can be actively developed in other world nations through IPSO network partnerships. Different nations (i.e., in your own country) may offer varied new opportunities. So regularly check it out! ….

## The Future of Pediatric Surgical Oncology Training—Where to Next?

In the UK currently there are too many pediatric surgery programmes (over 22 centers with a national population > 68 million) providing cancer services for children. As a result individual surgical centers manage and treat few index solid tumor malignancies to provide wholly sufficient training in pediatric surgical oncology with residents competing with consultant surgeons who now frequently share operating in teams to maintain skill competencies. Similar scenarios may exist in other world countries requiring a critical reappraisal with the considered future development of pediatric cancer surgery “super centers”. As an example in the Netherlands (national population 17 million) pediatric cancer surgery (2014 –) is now restricted to a single major center located at the Princess Maxima Center in Utrech, NL. A health care model such as this concept is considered “forward thinking” in vision to create a national center of excellence teamed and “tooled up” with a highly skilled infrastructure to sustain credibility.

Perhaps the same thoughts and ideas may be uttered for other world countries with their pediatric cancer health care systems. We must shift the imbalance. Without pediatric surgical oncologists and cancer specialists working together and sharing a leadership role in health care many of our hospitals seeking to deliver world class services will suffer. Surgical training programmes working toward national ranking(s) in residency and oncology fellowship applications should also “think smart” to be competitive. The drive toward “evidence based surgical oncology” health service provision, training and innovation(s) in cancer care have never been greater than in our current times. The best hospitals in the world will reassuringly always value well-trained inspirational pediatric surgical oncology leaders. I wish you all—“Good Luck”. . Get started now on your journey!.

“*A Career In Surgical Oncology Is Life Long Learning”…*

## Author's Note

PDL served as Chair UK CCLG Surgeons Group (2015–2017). PDL is currently President Elect - International Society Of Pediatric Surgical Oncology (IPSO).

## Data Availability Statement

The original contributions presented in the study are included in the article/supplementary material, further inquiries can be directed to the corresponding author/s.

## Author Contributions

The author confirms being the sole contributor of this work and has approved it for publication.

## Conflict of Interest

The author declares that the research was conducted in the absence of any commercial or financial relationships that could be construed as a potential conflict of interest.

## Publisher's Note

All claims expressed in this article are solely those of the authors and do not necessarily represent those of their affiliated organizations, or those of the publisher, the editors and the reviewers. Any product that may be evaluated in this article, or claim that may be made by its manufacturer, is not guaranteed or endorsed by the publisher.
